# Supplementary material for: AutoCryoPicker: an unsupervised learning approach for fully automated single particle picking in Cryo-EM images
Source: BMC Bioinformatics. 2019 Jun 13;20:326. doi: 10.1186/s12859-019-2926-y (PMC6567647; doi:10.1186/s12859-019-2926-y)
Supplement: Supplementary file 1 — Supplementary document. (DOCX 100 kb) [file 12859_2019_2926_MOESM1_ESM.docx]

# Additional file 1

**Algorithm 1: Intensity Based Clustering (IBC)**

Let $\left\{ I_{1},I_{2},\ldots,I_{n} \right\}$ be a set of images of the same modality containing the same anatomical structure of various subjects (i.e. particles in the cryo-EM images) and let $\left\{ x^{(1)},x^{(2)},\ldots,x^{(L)} \right\}$ be the set of all pixels in an image. Each pixel ($x^{l}$) will be grouped into several consistent “clusters” where the number of clusters is determined according to a specific intensity interval size. To determine the initial number of clusters in the ICB algorithm, for example $K=4$, if the adjusted intensity range is [0.2, 0.8] as shown in Figure 3(b) and the interval size is 0.15, there are 4 initial cluster levels: the intensity level [0.2-0.35] will be assigned to Cluster 1, [0.35-0.5] to Cluster 2, [0.5-0.65] to Cluster 3, and [0.65-0.8] to Cluster 4. Here, $x^{\left( i \right)}$ is a real intensity value in a specific range, $1 <= i <= L$. Let $\left\{ \theta_{1,}\theta_{2},\ldots,\theta_{K} \right\}$ be the set of the average intensity values of $K$ clusters. The centers $\left( \theta_{i},\theta_{j} \right)$ of $K$ clusters are initialized as evenly distributed intervals in the intensity range at the equal step size according to Equation (1):

$S_{size}=\frac{I_{Range}}{K\times0.15}\times0.15$ (1)

where the $I_{Range}$ is the difference between the maximum and the minimum intensity level in each image and 0.15 is the selected interval step size value.

Let $U_{j}$ be the index of the cluster whose center ($\theta_{i})$is closest to $x^{\left( i \right)}$. We denote the cluster assignment of all pixels after the desired centroid and cluster label ($C)$ is predicted as$\left\{ \langle x^{(1)},U_{1}\rangle,\langle x^{(2)},U_{2}\rangle,\ldots,\langle x^{(n)},U_{j}\rangle\right\}$. Each pixel $x^{(i)}$ is assigned to a specific cluster $k^{(j)}$ whose cluster center ($\theta_{k})$ is closest to the $x^{(i)}$ according to the absolute intensity difference value using Equation (2):

$C^{(i)}=\left| x^{(i)}-\theta_{k} \right|$ (2)

The final clusters centroids are updated iteratively according to the average intensity ${(\theta}_{j})$of clusters according to Equation (3):

$\theta_{j}=\frac{\sum_{i=1}^{m} \left\{ x^{(i)} \right\}}{\sum_{i=1}^{m} {\{L}^{(i)}=j\}}$ (3)

The procedure of the clustering algorithm for cryo-EM image clustering is shown below.

| **Algorithm 1** Intensity Based Clustering (IBC) |
| --- |
| 1. **input:** pre-processed cryo-EM image $I_{p}$ |
| 1. **return:** clustered image $I_{c}$ |
| 1. Initialize the minimum and maximum intensity mapping threshold values. |
| 1. Identify the cluster number $K$ to be 4 by mapping the input image intensity level to 4 levels based on the intensity adjusted min and max threshold values. |
| 1. Convert the 2-D image $I_{p}$into 1-D $I_{v}$which has the intensity values of all the pixels$.$ |
| 1. $L$← height ⋅ width where $L$ is the total number of pixels in the $I_{p}$ |
| 1. $V_{Max}$← $Max[I_{v}]$ /*maximum values of intensity in the image*/ |
| 1. $V_{Min}$← $Min[I_{v}]$ /*minimum values of intensity in the image*/ |
| 1. **for** $i$ = 1 to $K$ **do** |
| 1. $\theta_{k}\leftarrow{Int}_{s}\left[ i \right]$ /*Initialize the cluster centroids $\theta_{1},\theta_{2},\ldots,\theta_{K}\in R^{n}$ by computing the interval step size using Equation (8) */ |
| 1. **end for** |
| 1. **repeat** |
| 1. **for** $i$=1 to every intensity pixel $x^{(i)}$ **do** |
| 1. **for** $j$ = 1 to each cluster $K$ do |
| 1. Assign $x^{(i)}$ the cluster $k$ whose center ($\theta_{k})$ is closest to $x^{(i)}$ according to the absolute intensity difference using Equation (9). |
| 1. **end for** |
| 1. **end for** |
| 1. **for** $n$ = 1 to each cluster $K$ **do** |
| 1. Recompute the centroid of each cluster according to the average intensity ${(\theta}_{j})$of each cluster using Equation (3). |
| 1. **end for** |
| 1. **until** convergence, i.e. there is no change in cluster centres. |

**Algorithm 2: Image Cleaning and Non-Circular Object Removal**

An intermediate binary image $I_{Clustered}$is generated first to enlarge each object in the original binary clustered image by applying the morphological image operation on the original clustered image (binary) $C_{Image}$using image opening according to Equation (4)

$I_{Clustered}=\left( C_{Image}⊖S_{sub\_image} \right)\bigoplus S_{sub\_image}$ (4)

Where $I_{Clustered}$ is the original clustered image, $S_{sub\_image}$ is the structural sub image using circular structure $5\times5$, $⊖$ and $\bigoplus$ denote erosion and dilation respectively. Then, the small objects and non-circular ones are removed form the intermediate images based on the object roundness class which is determine by computing the area and perimeters using the connected component pixel index list and the circularity based on the Equation (5):

$Circularities=\frac{{allPerimeters}^{2}}{4\times pi\times allAreas}$ (5)

The image cleaning and small object removal algorithm is shown below.

| **Algorithm 2** Image Cleaning and Non-Circular Object Removal |
| --- |
| 1. **input:** $I_{c}$ /*cluster cryo-EM image */ |
| 1. **return:** $I_{cc}$ /*cleaned cluster image */ |
| 1. $I_{c1}\leftarrow imopen(I_{c})$ /* Generate an intermediate clustered image by enlarge the small object using the image opening according to Equation (4) */. |
| 1. $L\leftarrow bwlabel(I_{c1})$ /* Label each object in the cluster image using MATLAB function ($bwlabel$) */. |
| 1. **for** i=1 to $L$ **do** /* for each object in the intermediate clustered image*/ |
| 1. $I_{object}\leftarrow state(L(k))$ /* determine the connected components (objects) in the image, including a list of indexing pixel locations for each one using MATLAB function (regionprops) */. |
| 1. $I_{object}\leftarrow bwareaopen\left( state(L(k)) \right)$ /*remove the object that has not a fully connected edge using MATLAB function (bwareaopen)*/. |
| 1. **end for** |
| 1. ${obj}_{number}\leftarrow is member(I_{object})$ /*extract the number of object (particles)*/ |
| 1. $L\leftarrow bwlabel$ /*label each object (particle)*/ |
| 1. **for** $i$=1 to $L$ **do** /* for each object (particles) */ |
| 1. Do size filtering and roundness filtering |
| 1. $Areas \leftarrow[props.Area]$ /* Determine the region area of each connected component (object) using MATLAB function (region props('Area')) */ |
| 1. $Perimeters \leftarrow[props.Perimeter]$ /* Determine the region perimeters of each connected component (object) using MATLAB function (region props (Perimeter)) */ |
| 1. $Circularities \leftarrow allPerimeters^2/((4\times pi\times allAreas))$ /* Determine the region circularitiesof each connected component (object) using Equation (5). |
| 1. $Threshold_{area}\leftarrow50000$ /*determine the average objects "roundness" circularities value. */ |
| 1. $keeperObjects\leftarrow circularities < 3 \& Areas < threshold_{area}$ /* Keep objects that less than or equal to the average object’s "roundness" circularities value using MATLAB function (bwareaopen) */. |
| 1. Get actual index numbers instead of a logical vector |
| 1. $I_{c2}\leftarrow$produce new binary image with only the small, round objects in it |
| 1. $I_{cc}\leftarrow bwareaopen(I_{c})$ /*remove the object that has not a fully connected edge*/ |
| 1. **end for** |
| 1. Construct the output image containing only the object circular “roundness” object classes in each image. |

**Algorithm 3: Circular Hough Transformation (CHT)**

The CHT first generates a binary image based on each object’s edges. It then calculates the center and radius of each detected circular object. Each circular object in the binary image is defined by three parameters: the coordinates of the center $(a,b)$ and the radius $R$ as Equations (6) and (7) show [29]:

$x=a+R\times Cos\theta$ (6)

$y=b+R\times Sin\theta$ (7)

where $\theta$ ranges from 0 to ${360}^{^{\circ}}$, and $R$ is the radius.

In this case, CHT is looking for each circular object of a particular radius $(R)$ based on every boundary point $(p)$ in the clustered image (binary image) using the original coordinated system (xy) space as shown in Equations (6) and (7). Then, every point in the (xy) space is equivalent to a circle in the (ab) space by rearranging Equations (6) and (7) to form the following Equations (8) and (9).

$a=x_{1}-R\times Cos\theta$ (8)

$b=y_{1}-R\times Sin\theta$ (9)

Next, all $(R)$, indexed by theta, are retrieved from Hough space. For each of these R(\theta), a vote is placed in Hough space at $p + R( \theta)$. Finally, the cells that receive the greatest number of votes are selected as the centers of the circular objects. In most applications of CHT it is common to use Canny edge detection [29] for the construction of the binary map. In this application Canny edge detection fails to identify sufficient points for CHT to detect the circular object center (top-view particles) as is shown in Figure 10(b) and (e). To overcome this issue, the canny edge detection step is replaced by our IBC algorithm~~.~~ The pixels which makeup the outline of each circular object is extracted to form a boundary pixel list. This is done by removing interior pixels and then treating non-zero pixels as belonging to the object and considering zero valued pixels as the background. In our method a vector $P$, which contains two elements, is extracted by specifying the row and column coordinates of each point on the object boundary by tracing the 4-connected neighbors (setting them to 1). Each direction of the object boundary is traced to specify the direction of the object boundary. The result is a Q-by 2 matrix called the$B$ factor, where Q is the number of boundary pixels for the region.

The modified Circular Hough Transform algorithm (CHT) is shown below.

| **Algorithm 3** Circular Hough Transformation (CHT) |
| --- |
| 1. **input:** $I_{cc}$ /*cleaned cluster image */ |
| 1. **return:** number of circular object (particles) in the cryo-EM |
| 1. center $(x,y)$ of each circular particle, |
| 1. radius $r$ of each one. |
| 1. $P\leftarrow Trace[object]$ /* Determine and extract the boundary pixels list by specifying the row and column coordinates of each point on the object boundary */ |
| 1. Construct the output binary image containing only the object circular boundary for each object. |
| 1. /* *Hough Transform Begin**/ |
| 1. **for** $i=$1 to each edge point **do** |
| 1. Draw a circle with centre $(x,y)$ in the edge point with $r$ where $(x,y)$ is the image pixels with position $x,$ and $y$, $r$ is the circular radius. |
| 1. Increment all coordinates $(x,y)$ that the perimeter of the circle passes through in the accumulator. |
| 1. Find one or several maxima in the accumulator |
| 1. Map the found parameters $(r,a,b)$corresponding to the maxima back to the original image, where $a$, and $b$ is the centre of the maxima. |
| 1. **end for** |
| 1. /* *Hough Transform End**/ |

**Algorithm 4: Circular and Non-Square Object (Particles) Removal**

The main idea of the circular and non-square object removal is illustrated by two steps. First, each object (particle) in the cryo-EM (clustered image) is smoothed using a gaussian filter based on Equation (10) [29]:

$g\left( m,n \right)=G_{\sigma}(m,n)\times f\left( m,n \right)$ (10)

where $g\left( m,n \right)$ is the output smoothed image, $f(m,n)$ is the original input image (clustered), and $G_{\sigma}$is the gaussian kernel (mask) which is constructed based on using Equation (11) [29]:

$G_{\sigma}=\frac{1}{\sqrt{2{\pi\sigma}^{2}}}e^{\left( -\frac{m^{2}+n^{2}}{2\sigma^{2}} \right)}$ (11)

where $\sigma$ is the sigma (which represents the signal width), $m$, and $n$ is the image dimension. Next, a parallel of the image cleaning and non-circular object removal algorithm is constructed called the circular and non-square object (particles) removal. In this case, first we remove any other objects such as ice artifacts in the binary image (clustered) by removing any object that does not have a square shape. The square object shapes are determined by extracting the square object’s edges using the MATLAB function (*bwareaopen*). Then, instead of keeping all circular objects found in each region by Algorithm 2, objects with circularity value below the computed average are removed. Finally, we implement the reserve objective function of the Algorithm 2 to eliminate any other object that has region’s area size larger that the computed threshold value (average region size). This keeps the squares and removes the circular objects from the final mask (clustered image) of the cryo-EM. The circular and non-square object (particles) removal algorithm is shown below.

| **Algorithm 4** Circular and Non-Square Object (Particles) Removal |
| --- |
| 1. **input**: $I_{cc}$ /*cleaned cluster image */ |
| 1. **return**: $I_{cs}$ /*cleaned cluster image with square shapes only*/ |
| 1. $L\leftarrow bwlabel(I_{c1})$ /* Label each connected components (objects) in the cleaned image, including a list of pixel locations for each one using MATLAB function (bwlabel)*/ |
| 1. Generate a gaussian kernel (mask) using Equation (11). |
| 1. **for** each object in the cleaned clustered image **do** |
| 1. Smooth each object shape using gaussian filter according Equation (10) with specific kernel size=5x5. |
| 1. **end for** |
| 1. **for** $i$=1 to $L$ **do** /* **for** each object in the cleaned clustered image */ |
| 1. Remove the object that has not a fully connected edge using MATLAB function (bwareaopen) |
| 1. $allAreas\leftarrow[Area(L(i)]$ /* Determine the region area of each connected component (object) using MATLAB function (regionprops('Area')) */ |
| 1. $allPerimeter\leftarrow[Perimeter(L\left( i \right))]$ /* Determine the region perimeters of each connected component (object) using MATLAB function (regionprops (‘Perimeter’)) */ |
| 1. $circularities \leftarrow(object)$ /* Determine the region circularitiesof each connected component (object) using Equation (5) */ |
| 1. $keeperObjects\leftarrow circularities < 3 \& Areas$ /* Remove all the connected components that are bigger or equal to the average pixels */ |
| 1. **end for** |
| 1. Construct an intermediate image containing only the non-circular object “roundness” in each image. |
| 1. **for** $i$=1 to $L$ **do** /* **for** each object in the intermediate clustered image */ |
| 1. Determine the region area of each connected component (object) using MATLAB function (*regionprops('Area')*) |
| 1. $Max\_Allowable\_Area\leftarrow Max(Area(i))$ /* find the max area for all objects*/ |
| 1. **end for** |
| 1. **for** each object in the intermediate clustered image **do** |
| 1. Determine the region area of each connected component (object) using MATLAB function (*regionprops('Area')*) |
| 1. Determine the region perimeters of each connected component (object) using MATLAB function (*regionprops(‘Perimeter’))* |
| 1. Determine the region circularitiesof each connected component (object) using Equation (5). |
| 1. **If** $circularities$ < $Max\_Allowabl\_Area$ **then** |
| 1. $RoundObjects\leftarrow circularities < 3 \& Max\_Allowable\_Area$/* Keep objects that less than the maximum allowable area using MATLAB function (bwareaopen)*/ |
| 1. ${obj}_{position}\leftarrow is member(I_{object})$ /*Extract each object position using MATLAB function (*ismember*)*/ |
| 1. **end if** |
| 1. **end for** |
| 1. $I_{cs}\leftarrow ismember\left( I_{cc}, RoundObjects \right)> 0$ /* Construct the cleaned output image containing only the squarest object classes occurring in each image */ |

**Algorithm 5: Square (Side View) Particle Detection and Picking**

The particle detection and picking algorithm based on first determining the connected components (for each objects) in the cleaned cryo-EM image, including a list of pixel area and locations for each one. Second, since some of the artifact objects have a fully connected component and almost the same size the side view particles, we determine and extract the smallest rectangle region area. Third, we determine the region area of each connected component (object) and keep the objects that are less than the smallest rectangle region area. Finally, bounding boxes are drawn around each discontinuous region (rectangle region area) after determining the connected components (objects) in the image. A list of pixel locations for each object is returned along with the extracted centroid, defined by the horizontal and vertical coordinates (x,y). The square (side-view) particle detection and picking is shown below.

| **Algorithm 5** Square (Side View) Particle Detection and Picking |
| --- |
| 1. **input**: $I_{cs}$ /*cleaned cluster image with square shapes only*/ |
| 1. **return**: $I_{cps}$ /*cleaned cluster image with perfect square shapes*/ |
| 1. $L\leftarrow bwlabel(I_{c1})$ /* Label each connected components (objects) in the cleaned image and extract the total number of objects including a list of pixel locations for each one using MATLAB function (bwlabel)*/ |
| 1. **for** i=1 to $L$ **do** /* for each object in the cleaned clustered image*/ |
| 1. $Stats \leftarrow regionprops\left( I_{cs} \right)$ /* Determine the class measure properties of each connected component using MATLAB function (regionprops). */ |
| 1. $Areas \leftarrow[props.Area$] /* Determine the region area of each connected component (object) using MATLAB function (*regionprops('Area')*) l*/ |
| 1. ${Min}_{area}\leftarrow min[Areas(i)]$ /* Determine and extract the smallest rectangle region area */. |
| 1. $keeperObjects\leftarrow Areas< {Min}_{area}$ /*keep objects that are less that the smallest rectangle region area. */ |
| 1. **end for** |
| 1. **for** $i$=1 to $size(keeperObjects)$ **do** /* for each rectangle region area. in the cleaned clustered image */ |
| 1. $\left[ x,y \right]\leftarrow centroid\left( keeperObjects \right)$ /*Determine the connected components (objects) in the image, including a list of pixel locations for each one and extract the centroid is the horizontal coordinate (or x-coordinate) and vertical coordinate (or y-coordinate) using MATLAB function (*regionprops('centroid’*)) */. |
| 1. Draw all bounding box for each discontinuous region (rectangle region area). |
| 1. **end for** |

**Algorithm 6: Perfect Square Particles Shape Detection and picking**

The perfect square particles shape detection and picking has three main steps. The first step removes the small attached objects by smoothing each particle. Each particle is convolved with a 50x50 averaging filter kernel. The main particles smoothing (averaging) can be defined in Equation (12) [29]:

$C\left( i,j \right)=\sum_{p} \sum_{q} A\left( p,q \right)B(i-p+1,j-q+1)$ (12)

where $C\left( i,j \right)$ is defined as each particle sub image, $B$ is the smoothing (averaging) kernel, $p$ and $q$ are the particle sub image dimensions. In the second step, after the small attached object are removed for each particle, we use the Feret diameter measures approach [37] to measure and correct the particle object dimensions. New perfect particle shapes are generated based on the maximum and the minimum Feret diameter. The maximum and minimum dimensions (width) of the particle object are used to identify the antipodal vertex pairs from the convex hull vertices set. Based on the new boundary box dimension, new perfect shapes are generated and inserted above each particle in the clean clustered cryo-EM image. The last step eliminates the outliers object (overlapped particles) by defining the average particles size and eliminating the outliers that have particle size larger than the average size. Then, the new boundary box is drawn based on the dimension of new particle object shapes. The perfect square particles shape detection and picking is shown below.

| **Algorithm 6** Perfect Square Particles Shape Detection and picking |
| --- |
| 1. **input:** $I_{cps}$ /*cleaned cluster image with perfect square shapes*/ |
| 1. ${Kernek}_{size}\leftarrow5\times5$ |
| 1. **return**: number of perfect square particles |
| 1. $I_{cps}$ /*cleaned cluster image with perfect square shapes*/ |
| 1. $L\leftarrow bwlabel(I_{c1})$ /* Label all connected components (objects) in the cleaned image and extract the total number of objects including a list of pixel locations for each one using MATLAB function (*bwlabel*)*/. |
| 1. **for** each object in the cleaned clustered image **do** |
| 1. $I_{smoothed}\leftarrow Smooth(I_{object},{Kernek}_{size})$ /* remove the small object by smoothing each object shape using Equation (10) with specific kernel size=5x5 */. |
| 1. **end for** |
| 1. **for** $i$=1 to each object (particle object) $L$ **do /*** each object in the cleaned clustered image*/ |
| 1. $I_{object}\leftarrow state(L(k))$ /* get each particle where $k$ is the total number of objects in the cluster cryo-EM*/ |
| 1. $L\leftarrow bwlabel(I_{smoothed})$ /*measure set of properties specified by properties for each 8-connected component in the binary image using MATLAB function (*regionprops)* */. |
| 1. Calculate the ferret properties |
| 1. **for** $i$=1 to each pixel in the cleaned binary mask **do** |
| 1. $P_{list}\leftarrow PixelList(objects)$ **/***convert each object pixel to coordinates as an x-y order including a list of pixel locations for each one using MATLAB function (*PixelList*)*/ |
| 1. $P_{hull}\leftarrow PixelHull(P_{list})$ /*extract the pixel hull diamond shapes using MATLAB function (*PixelHull*) */ |
| 1. $P_{pairs}\leftarrow VerticPair(P_{hull})$ **/***Determine the maximum Feret diameter and its orientation (maximum diameter) */ |
| 1. ${Feter}_{dimeter}\leftarrow Min(P_{pairs})$ /*computes the minimum ferret diameter*/ |
| 1. ${Area}_{bounding}\leftarrow Min\left( {Feter}_{dim} \right)$/*extract the minimum bounding box area*/ |
| 1. **end for** |
| 1. $P_{list}\leftarrow PixelList(objects)$ **/***Convert each object pixel to coordinates as an x-y order including a list of pixel locations for each one using MATLAB function (*PixelList*)*/ |
| 1. **for**$i$=1 to size(objects) **do** /* each object (particle) in the cleaned binary mask*/ |
| 1. Extract the bounding box dimension |
| 1. Extract the 2D convex hull of the points $(X,Y)$ for each object (particle) /*$X$ and $Y$ are column-vectors which presents a vector of point indices arranged in a counter-clockwise cycle around the hull */ |
| 1. **end for** |
| 1. $I_{s}\leftarrow Insert\left( {Area}_{bounding}(x,y) \right)$ /*Generate the final image with perfect squares shape generation*/ |
| 1. $L\leftarrow bwlabel($ /*find the connected components of all objects (particles) in binary image*/ |
| 1. $Stats \leftarrow regionprops\left( L \right)$ /* measure properties of particle region*/ |
| 1. determine and eliminate the outliers object (particles) |
| 1. **for** $i$=1 to $L$ **do** /* each object in the binary image*/ |
| 1. $Avearge\_area\leftarrow aveage(area(L(i)))$ /* find the average object area in binary image*/ |
| 1. **end for** |
| 1. **for** $i$=1 to $L$ **do** /*for each object in the binary image*/ |
| 1. **if** each $particle\_area$ <= $avearge\_area$ **then** |
| 1. keep this object by getting the actual index numbers instead of a logical vector. |
| 1. $I_{object}\leftarrow particle\_area$ <= $avearge\_area$ /* keep objects that are less that the average rectangle region area */ |
| 1. ${obj}_{postion}\leftarrow is member(I_{object}$ /* Extract each object position using MATLAB function (*ismember*)*/. |
| 1. **end if** |
| 1. **end for** |
| 1. Determine the connected components (objects) in the image, including a list of pixel locations for each one and extract the centroid is the horizontal coordinate (or x-coordinate) and vertical coordinate (or y-coordinate) using MATLAB function (*regionprops('centroid’*)). |
| 1. Draw all bounding boxes for each discontinuous region (rectangle region area). |
